# A Homemade Snare: An Alternative Method for Mechanical Removal of* Dirofilaria immitis* in Dogs

**DOI:** 10.1155/2016/5780408

**Published:** 2016-02-11

**Authors:** Ana Margarida Alho, António Fiarresga, Miguel Landum, Clara Lima, Óscar Gamboa, José Meireles, José Sales Luís, Luís Madeira de Carvalho

**Affiliations:** ^1^CIISA, Faculty of Veterinary Medicine, ULisboa, Avenida da Universidade Técnica, 1300-477 Lisboa, Portugal; ^2^Cardiology Unit, Hospital de Santa Marta, Centro Hospitalar de Lisboa Central, EPE, Rua de Santa Marta 50, 1169-024 Lisboa, Portugal; ^3^Small Animal Teaching Hospital, Faculty of Veterinary Medicine, ULisboa, Avenida da Universidade Técnica, 1300-477 Lisboa, Portugal

## Abstract

Canine dirofilariosis is a life-threatening parasitic disease that is increasingly reported worldwide. Once diagnosed the main treatment goals are to improve the animal's clinical condition and to eliminate all life stages of the parasite with minimal posttreatment side effects. This can be achieved through mechanical, surgical, or chemotherapeutical approaches. Currently, manual extraction is the preferred method to remove adult heartworms due to its diminished invasiveness, reduced damage to the vascular endothelium, and shortened anaesthesia duration. However, it remains an expensive technique that can be highly traumatic. To address this issue, a nontraumatic homemade catheter-guided snare was developed for heartworm removal by adapting and folding a 0.014-inch coronary wire (BMW, Abbott Vascular). Transvenous heartworm extraction was performed on a dog severely infected with adult heartworms by inserting the modified snare into a 6-F Judkins right coronary guiding catheter BMW (Cordis) and advancing it into the right ventricle under fluoroscopic guidance. Fifteen adult specimens of* Dirofilaria immitis* were successfully extracted from the pulmonary artery and right ventricle without complications. To assure the death of both larvae and adults, postoperative treatment was successfully managed using ivermectin, doxycycline, and melarsomine, with no recurrence after surgery.

## 1. Introduction

Canine dirofilariosis is a severe canine vector-borne disease with potentially fatal consequences. It is widely distributed throughout the world, with an increasing incidence in previously nonendemic areas [1, 2]. The main treatment goals are to improve the animal's clinical condition and to eliminate all forms of the parasite (microfilariae, larval stages, juveniles, and adults) with minimal complications. This can be achieved pharmacologically by combining melarsomine dihydrochloride, macrocyclic lactones, and doxycycline [3]. However, this approach can lead to several complications and adverse effects including pulmonary thromboembolism due to the worm death and anaphylactic shock secondary to the sudden death of high microfilariae counts [4]. For this reason, either mechanical or surgical heartworm removal is generally preferred as a means to eliminate as many adult worms as possible before pharmacological treatment is initiated. Manual extraction is the preferred method due to its diminished invasiveness, reduced damage to the vascular endothelium, and shortened anaesthesia duration [4, 5]. However, it remains an expensive technique out of the reach of many owners. Additionally, some of the available devices are also traumatic. To address these issues a nontraumatic intravascular snare was developed by adapting an economical coronary wire (commonly used in human patients) to attempt heartworm removal.

## 2. Materials and Methods

### 2.1. Case Presentation

A senior unneutered mixed-breed male dog (body weight: 6.1 kg) was presented to the Small Animal Teaching Hospital of the Faculty of Veterinary Medicine, ULisboa, with a history of severe cough, weakness, dyspnoea, exercise intolerance, and syncope. The owner reported a recent episode of hind limb weakness and temporary loss of balance which lasted for approximately 40 seconds. The dog was adopted from a shelter one month prior to presentation and his age was unknown (10 years old approximately). During the intervening period between adoption and presentation at the hospital no prophylactic treatment was initiated by the owner. On physical examination the dog had normal weight and was alert and responsive, but it was tachypnoeic and slightly dyspnoeic. Mucous membranes were pink with a capillary refill time of less than two seconds. Thoracic auscultation revealed an increased respiratory effort associated with mild crackles. A loud systolic regurgitant heart murmur (grade III approximately) was audible on the right side of the thorax, more significantly over the tricuspid valve and near the right side of the heart apex. The remainder of the physical examination was unremarkable.

### 2.2. Diagnostic Methods

Blood was collected from the cephalic vein and direct smears were performed. Under light microscopy, several microfilariae were observed. These were identified as* Dirofilaria immitis* using Knott modified test based on their morphometric characteristics [6]. The commercial WITNESS® Dirofilaria kit (Synbiotics Corp., Europe) was also used, supporting the previous diagnosis. A complete blood count was performed [white blood cell count, 11.3 × 10^3^/*μ*L (6–17 × 10^3^/*μ*L); red blood cell count, 6.2 × 10^6^/*μ*L (5.5–8.5 × 10^6^/*μ*L); platelet count, 347 × 10^3^/*μ*L (200–500 × 10^3^/*μ*L); haemoglobin, 14.1 g/dL (12–18 g/dL); haematocrit, 46.8% (37–55%); eosinophils, 1.4 × 10^3^/*μ*L (0.1–1.3 × 10^3^/*μ*L)], revealing a mild eosinophilia. Routine serum biochemistry profile was also performed [glucose, 107 mg/dL (60–125 mg/dL); total protein, 9.0 g/dL (5.1–7.8 g/dL); creatinine, 0.7 mg/dL (0.4–1.8 mg/dL); alkaline phosphatase, 116 *μ*l/L (10–150 *μ*l/L)]. Prerenal azotaemia [blood urea nitrogen, 52.3 mg/dL (7–27 mg/dL)] and moderately increased hepatic enzymes, alanine transaminase (ALT) [240 *μ*l/L (5–60 *μ*l/L)] and aspartate transaminase (AST) [96.6 *μ*l/L (5–55 *μ*l/L)], were found, possibly explained by passive liver congestion due to right cardiac overload.

**Figure 1:**
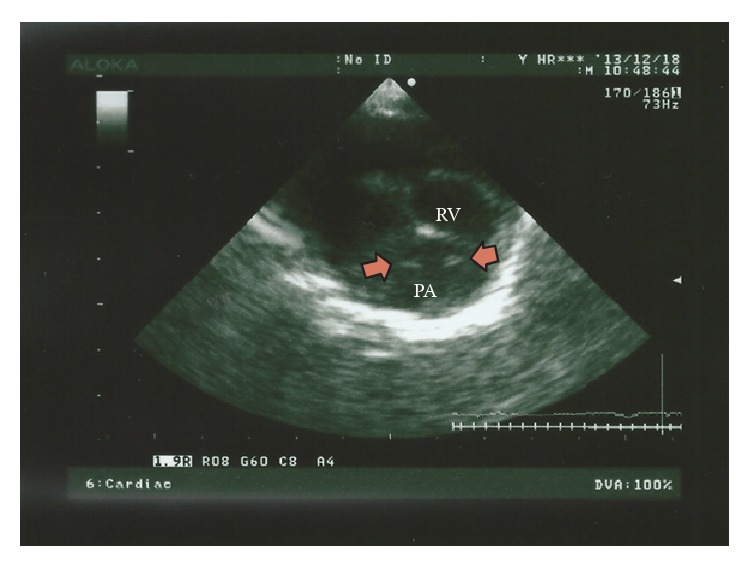
An echocardiographic image of the right ventricle (RV) and pulmonary artery (PA), in a short axis view, right parasternal section, in a right lateral decubitus. Note the presence of linear, parallel hyperechoic structures corresponding to adult worms (arrows) within the pulmonary artery.

To assess the severity of heartworm cardiopulmonary disease, lateral and ventrodorsal radiographic projections of the thorax were made at full inspiration, revealing slight dilation of the right ventricle and bulging of the pulmonary arteries. Vertebral heart score was 9.8 (8.7–10.7) and there was no evidence of lung inflammation in the areas surrounding the pulmonary arteries. Further transthoracic echocardiography revealed the presence of linear, mobile, parallel hyperechoic structures (short parallel-sided images with the appearance of “equal signs”) within the right ventricle outflow tract and main pulmonary artery, consistent with the presence of heartworms (Figure 1). Spectral Doppler echocardiography showed mild tricuspid regurgitation (velocity of 2.3 meters per second). Additionally, slight dilation of the right ventricle was noticed without increase of the pulmonary flow velocity or abnormal tricuspid relation between E wave and A wave [E : A ratio]. No heartworms were visualized within the tricuspid orifice or posterior vena cava, excluding the diagnosis of caval syndrome.

Considering the clinical signs exhibited by the dog (coughing, exercise intolerance, weakness, dyspnoea, and syncope), the abnormal findings on the thoracic radiography, and the visualization of hyperechoic structures consistent with parasites within the right ventricle and pulmonary artery, it was concluded that the dog was severely infected with heartworms [3] and was at high risk for thromboembolic complications. As overall survival is significantly improved in animals that undertake mechanical heartworm removal (prior to the adulticide therapy) [3], and as the echocardiography showed worms in accessible locations to be percutaneously removed, heartworm removal procedure was proposed using a homemade snare. Owner's informed consent was given and heartworm removal was scheduled.

### 2.3. Transvenous Heartworm Extraction Procedure

Two weeks prior to surgery the dog was stabilized with furosemide (1 mg/kg, per os [PO], twice a day [BID]) and enalapril (0.5 mg/kg, PO, once a day [SID]) to minimize cardiac overload. Also doxycycline (10 mg/kg, PO, BID) was prescribed at the same time. In order to reduce the thromboembolic risk associated with heart catheterization and adult worm death, the dog was also started on prednisolone (0.5 mg/kg, PO, BID) and cetirizine (1 mg/kg, PO, SID) one week prior to the procedure.

**Figure 2:**
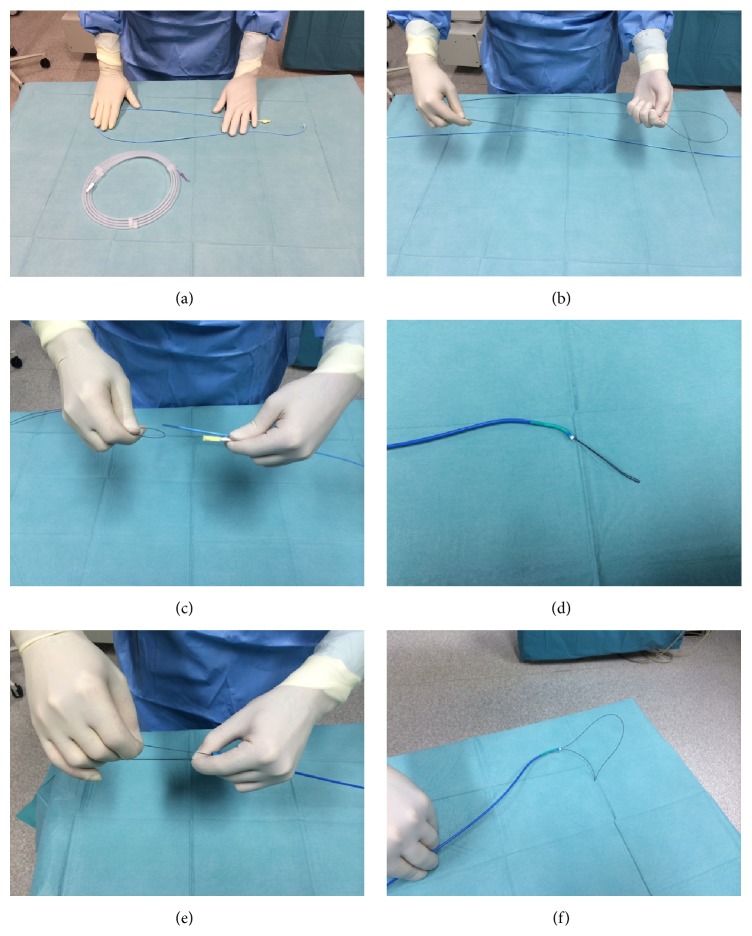
Mechanical heartworm removal device used during the procedure. (a) A snare introducer, a 6-F plastic sheath, inserted via the right external jugular vein. (b) A specific carrier, a 6-F Judkins right coronary guiding catheter BMW (Cordis). ((c), (d), and (e)) A 0.014-inch coronary wire (Boston Scientific) that was folded and pushed through the coronary guiding catheter. (f) Final aspect of the homemade snare.

**Figure 3:**
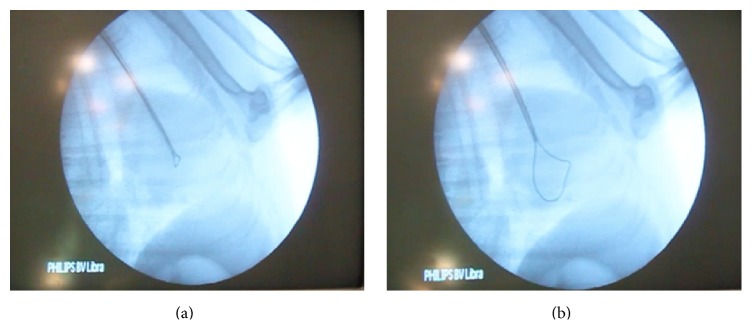
Heartworm surgical extraction under fluoroscopy guidance. (a) A 6-F Judkins guiding catheter BMW (Cordis) and the loop wire, placed at the right ventricle. (b) Increasing the size of the loop wire in order to snare the heartworms, followed by gentle retraction of the snare.

On the day of the procedure, the dog was premedicated with heparin (100 U/kg, subcutaneously [SC]) and an association of amoxicillin and clavulanic acid (20 mg/kg, BID, intramuscularly [IM]). Anaesthesia was induced with propofol (4 mg/kg, intravenously [IV]) and maintained with isoflurane (2–2.5% concentration) after tracheal intubation. The dog was kept in left lateral recumbence and the right side of the cervical region was prepared. Venous puncture was performed using the Seldinger technique and a 6-F plastic sheath was introduced via the right external jugular vein. Anticoagulation was enhanced with intravenous heparin (100 U/kg). Under fluoroscopic guidance, a 6-F Judkins right coronary guiding catheter BMW (Cordis) was introduced and moved towards the cranial vena cava, right atrium, and right ventricle. A homemade snare was created by folding a 0.014-inch coronary wire (Boston Scientific) (Figure 2). This device was subsequently inserted into the guiding catheter keeping both distal parts exteriorized. The operator fixed one of the wire extremities with one hand and moved the other end forward, adapting the size and shape of the loop according to the number and location of the worms (Figure 3). To retract the worms through the catheter, both extremities of the wire were gently withdrawn at the same time.

Since navigation into the pulmonary artery was difficult with the 8-F guiding catheter, it was downsized to a 6-F model. For this reason, a smaller device was created using 0.014-inch coronary wire (BMW, Abbott Vascular), which was folded using the same method described previously. This homemade snare was then used to pull out the remaining heartworms through the sheath.

## 3. Results

**Figure 4:**
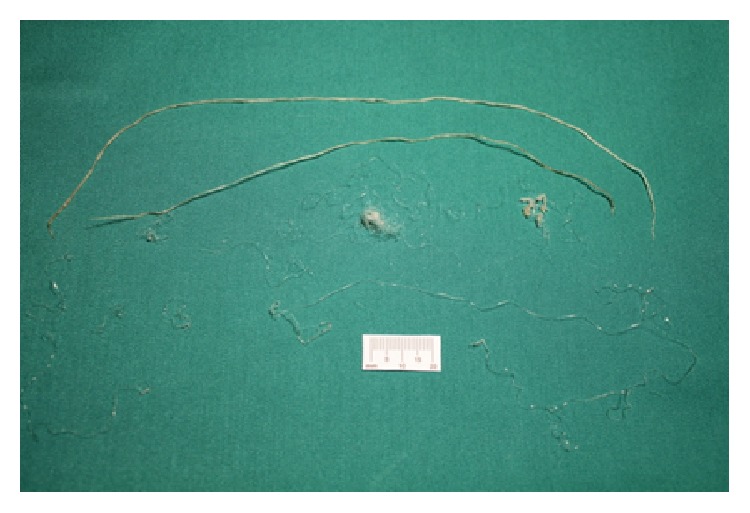
Retracted worms. Note the 15 specimens of* Dirofilaria immitis* extracted with the homemade snare from the right side of the heart and pulmonary artery.

In total, fifteen adult specimens of* Dirofilaria immitis* were caught and gently retracted through the catheter from the right ventricle and proximal portion of the pulmonary artery (Figure 4). Considering the risks of cardiac arrest and potential heart and vascular lesions due to continued catheter manipulation as well as the prolonged duration of the anaesthesia, the catheter was retracted and no further attempts were made. Haemostasis was achieved with manual compression and the dog was sent to the intensive care unit after the procedure was completed. Recovery occurred without complications and the dog was discharged after careful evaluation with amoxicillin and clavulanic acid (20 mg/kg, BID, PO) and with instructions for the owner to restrict exercise.

A postoperative reevaluation was scheduled seven days after the procedure. Once the dog was recovering well, treatment with ivermectin (10 *μ*g/kg, PO) was initiated to prevent potential residual infection. Doxycycline (10 mg/kg, PO, for 28 days, BID) was also restarted. The first melarsomine injection was performed 60 days after surgery (2.5 mg/kg, IM). The second and third consecutive treatments were performed 90 and 91 days after surgery, as recommended by the American Heartworm Society [3]. Exercise restriction was imposed during the entire treatment regimen.

Three months after surgery, the dog was reevaluated. Clinical signs relating to the presence of heartworms were resolved and no murmurs were auscultated. The owner reported that the dog had a good appetite and energy levels but still coughed occasionally. Routine heartworm prevention on a monthly basis was recommended. Eight months after surgery, the dog was very alert and active and no coughing was reported. An additional commercial antigen, WITNESS Dirofilaria kit, was performed, testing negative for* D. immitis* infection.

## 4. Discussion and Conclusion

In order to offer an affordable and safe treatment to every owner, in cases where mechanical heartworm removal is the most appropriate treatment, a catheter-guided technique using a homemade snare for adult heartworms retrieval was developed.

In general, mechanical extraction is a far less invasive and painful method when compared to cardiothoracic surgery, allowing a faster recovery and reducing the risk of infection. The snare is a safer technique when compared with forceps or the horsehair brush, since it minimizes accidental intracardiac and vascular damage, frequently associated with blind grasping [7]. The snare is also advantageous in comparison with the basket retrieval device, since the operator can control the degree of closure of the snare and thus reduce the risk of traumatizing or breaking the ensnared worms [7]. The snare's loop can also be manipulated to adopt the size, shape, or angle intended by the practitioner, increasing the likelihood of worm retrieval. In addition, since venotomy is not required to access the jugular vein, surgical closure is not necessary and the subsequent bleeding associated with catheter insertion is practically insignificant. The snare also appears to be more effective over previously described heartworm extraction methods, namely, Ishihara and flexible alligator forceps, whose size only permits their introduction into the right atrium and proximal portion of the right ventricle and not through the tricuspid and pulmonic valves [7]. Furthermore, this homemade snare is less expensive than the specific snare usually employed for this task, since it only requires a sheath, a coronary guiding catheter, and a common coronary wire.

Despite the abovementioned advantages, general anaesthesia, fluoroscopic guidance, subsequent chemotherapy, and a skilled practitioner are still required [4, 7]. Besides, the potential risk for cardiac arrest and ventricular arrhythmias caused by snare manipulation in the right ventricle or even the risk of transecting an adult heartworm still remains. Without direct visualization of the worms, the success of percutaneous heartworm extraction will always rely upon the operator's ability to ensnare the worms, which is dependent on their anatomical location and burden and the size of the parasites. To accomplish a more efficacious heartworm extraction, care must be taken to move one of the snare tips while the other is maintained in a fixed position, in order to achieve the necessary loop size.

Scant data is currently available in the literature regarding transvenous procedures for adult heartworm retrieval in companion animals. The most common reported devices used are Ishihara forceps, Jones forceps, the horsehair brush, tripod forceps [8], basket forceps [8, 9], alligator forceps [10–13], endoscopic grasping forceps, flexible three wires nail tipped forceps [14], and the gooseneck snare [7]. More sophisticated commercial snares, which include the nitinol gooseneck snare, have total and reproducible memory allowing the loop to return to a specific shape and diameter, a considerable advantage over the homemade snare. But, evidently, these are more expensive and thus are not a viable alternative for low-cost surgery [15].

Further surgical transvenous interventions need to be done to validate and improve the efficiency of this technique. Nevertheless, we believe that the possible cost reductions and less traumatic damage induced by this snare, when compared to existing alternatives, will allow adult heartworm extraction to be more affordable and consequently widespread, thereby promoting the treatment of a larger number of animals, enhancing a specific chemotherapy with higher safety.

## Abbreviations

ALT: Alanine transaminase
AST: Aspartate transaminase
BID: Twice a day
E : A ratio: Relation between E wave and A wave
IM: Intramuscularly
IV: Intravenously
PO: Per os
SC: Subcutaneously
SID: Once a day.
